# Supplementary material for: Low-Molecular-Weight Heparin Reduces Ventilation-Induced Lung Injury through Hypoxia Inducible Factor-1α in a Murine Endotoxemia Model
Source: Int J Mol Sci. 2020 Apr 28;21(9):3097. doi: 10.3390/ijms21093097 (PMC7247708; doi:10.3390/ijms21093097)
Supplement: Supplementary file 1 [file ijms-21-03097-s001.pdf]

Low-Molecular-Weight Heparin Reduces Ventilation-Induced Lung Injury through Hypoxia Inducible Factor-1α in a Murine Endotoxemia Model

Li-Fu Li[1,2,3], Yung-Yang Liu[4,5], Shih-Wei Lin[1,2,3], Chih-Hao Chang[1,2], Ning-Hung Chen[1,2,3], Chen-Yiu Hung[1,2] and Chung-Shu Lee[1,2]

The following data are supplementary material

Bronchoalveolar lavage fluid macrophage inflammatory protein-2 and vascular endothelial growth factor protein production and oxidative loads were measured to determine the effects of low-tidal-volume mechanical ventilation (MV) with or without enoxaparin administration. No substantial differences of inflammatory cytokines and oxidative loads were observed between low-tidal-volume ventilated mice and low-tidal-volume ventilated mice receiving lipopolysaccharide with or without administration of enoxaparin (Figure S1). Because different animals have different respiratory mechanics, the physiologically valid magnitudes of mechanical ventilation for the developments of microvascular leak differ depending on the species of animals. In our mouse model of ventilator-induced lung injury, we used tidal volume 30 mL/kg based on the result of previous study that the nature of mouse respiratory mechanics is more compliant than human lungs and tidal volume up to 20 mL/kg are unlikely to induce substantial lung overstretch in models using healthy mice [1].

1. Wilson, MR.; Patel, B.V.; Takata, M. Ventilation with "clinically relevant" high tidal volumes does not promote stretch-induced injury in the lungs of healthy mice. *Crit. Care. Med.* **2012**, 40:2850-2857.

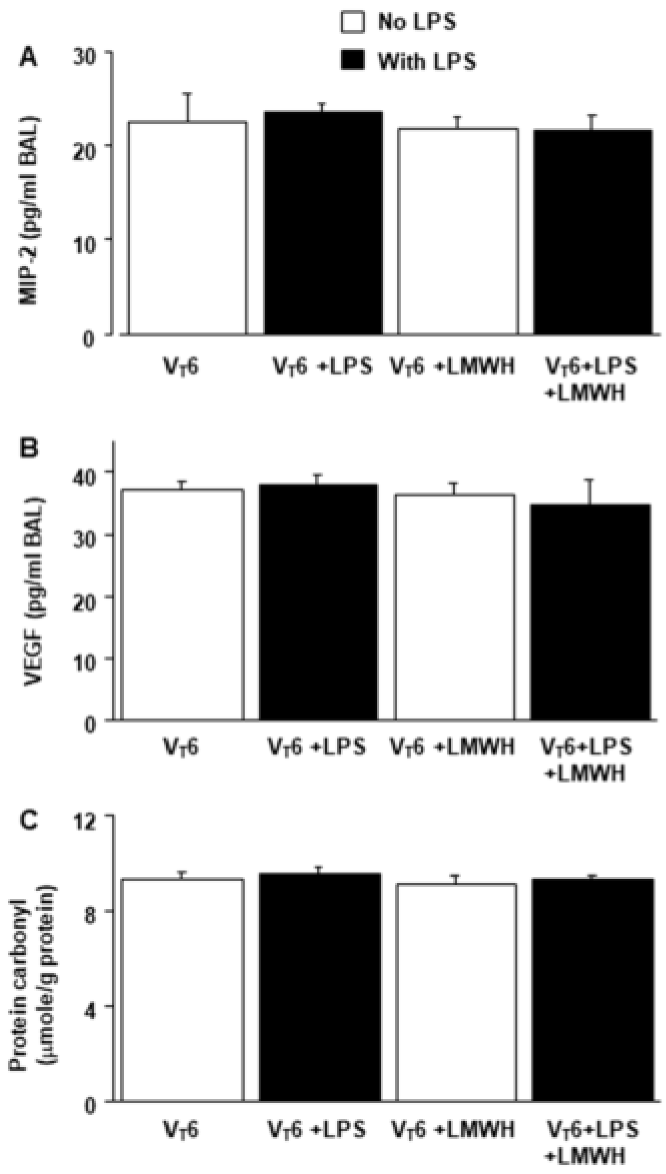

Figure S1. Effects of enoxaparin on endotoxin-aggravated low-tidal-volume mechanical ventilation-enhanced lung inflammation. BAL fluid total protein, (**A**) MIP-2 and (**B**) VEGF secretion and (**C**) protein carbonyl groups were from the lungs of mice subjected to a tidal volume of 6 mL/kg with or without LPS and enoxaparin administration (n = 3 per group). Enoxaparin, 4 mg/kg, was given subcutaneously 30 minutes before mechanical ventilation. BAL = bronchoalveolar lavage; LMWH = low molecular weight heparin; LPS = lipopolysaccharide; MIP-2 = macrophage inflammatory protein-2; VEGF = vascular endothelial growth factor; V_T = tidal volume.

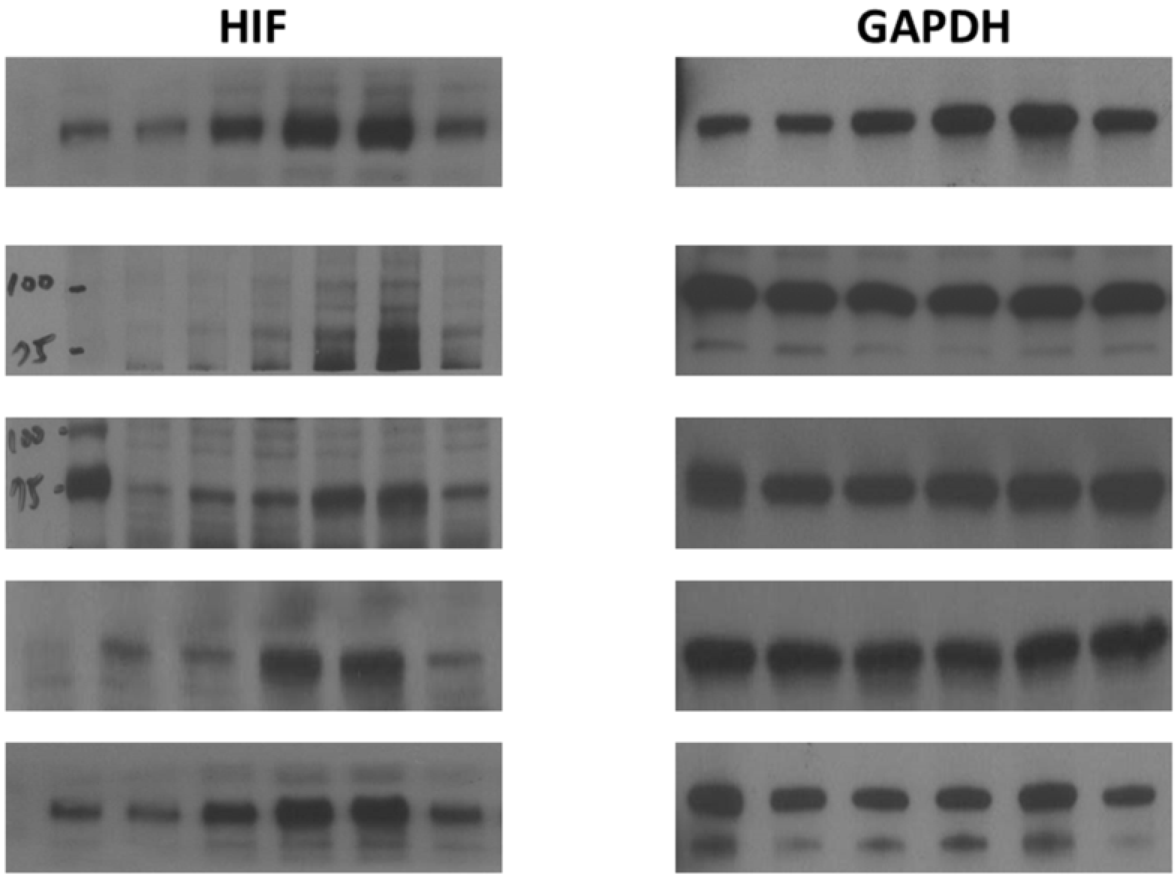

Figure 2. Original figure of Figure 4, left column: HIF; right column: GAPDH. HIF = hypoxia-inducible factor; GAPDH = glyceraldehydes-phosphate dehydrogenase.

Table 1. Physiologic conditions at the beginning and end of ventilation. .

	Nonventilated	Nonventilated LPS	V_T 6 ml/kg LPS	V_T 30 ml/kg LPS	V_T 30 ml/kg LPS+HIF-1α	V_T 30 ml/kg LPS+LMWH
PH	7.42 ± 0.05	7.39 ± 0.02	7.36 ± 0.08	7.39 ± 0.04	7.38 ± 0.05	7.38 ± 0.05
PaO2 (mmHg)	98.3 ± 0.2	92.2 ± 0.3	89.6 ± 0.5 *	73.5 ± 3.1 *	86.1 ± 2.4 *	85.4 ± 2.1 *
PaCO2 (mmHg)	39.2 ± 0.3	39.7 ± 0.2	38.7 ± 1.4	38.4 ± 1.5	37.5 ± 1.2	37.8 ± 1.4
MAP (mmHg)						
Start	85.4 ± 1.3	83.8 ± 0.6	84.9 ± 1.3	82.6 ± 2.8	84.8 ± 2.3	84.9 ± 2.5
End	85.0 ± 0.5	81.1 ± 0.3	79.1 ± 2.2 *	75.5 ± 2.3 *	78.2 ± 2.5 *	78.1 ± 2.7 *
PIP (mmHg)						
Start			15.9 ± 1.2	16.2 ± 1.4	15.8 ± 1.2	15.9 ± 1.4
End			16.8 ± 1.7	17.5 ± 1.9	17.2 ± 1.3	17.3 ± 1.6

At the end of the study period, we obtained data of mean arterial pressure and arterial blood gases from the nonventilated control mice and mice ventilated at a tidal volume of 6 or 30 mL/kg for 5 h (*n* = 10 per group). The normovolemic statuses of mice were maintained by monitoring mean artery pressure. Data are presented as means ± SDs. * indicates that $p < 0.05$ when compared to the nonventilated control mice. HIF = hypoxia-inducible factor; LMWH = low-molecular-weight heparin; LPS = lipopolysaccharide; MAP = mean arterial pressure; PIP = peak inspiratory pressure; V_T = tidal volume.
